# Arbuscular Mycorrhizal Fungi and Plant Chemical Defence: Effects of Colonisation on Aboveground and Belowground Metabolomes

**DOI:** 10.1007/s10886-017-0921-1

**Published:** 2018-02-02

**Authors:** Elizabeth M. Hill, Lynne A. Robinson, Ali Abdul-Sada, Adam J. Vanbergen, Angela Hodge, Sue E. Hartley

**Affiliations:** 1 School of Life Sciences, University of Sussex, Brighton, BN1 9QG UK; 2 Centre for Ecology and Hydrology (CEH), Bush Estate, Penicuik, Midlothian, EH26 0QB UK; 3 Department of Biology, University of York, Wentworth Way, York, YO10 5DD UK; 4 York Environment and Sustainability Institute, Department of Biology, University of York, Wentworth Way, York, YO10 5DD UK

**Keywords:** Arbuscular mycorrhizal symbiosis, Above-belowground interactions, Phytobiome, Microbiome, Metabolomics, Pyrrolizidine alkaloids, Blumenols, *Senecio jacobaea*, *Rhizophagus irregularis*

## Abstract

Arbuscular mycorrhizal fungal (AMF) colonisation of plant roots is one of the most ancient and widespread interactions in ecology, yet the systemic consequences for plant secondary chemistry remain unclear. We performed the first metabolomic investigation into the impact of AMF colonisation by *Rhizophagus irregularis* on the chemical defences, spanning above- and below-ground tissues, in its host-plant ragwort (*Senecio jacobaea*). We used a non-targeted metabolomics approach to profile, and where possible identify, compounds induced by AMF colonisation in both roots and shoots. Metabolomics analyses revealed that 33 compounds were significantly increased in the root tissue of AMF colonised plants, including seven blumenols, plant-derived compounds known to be associated with AMF colonisation. One of these was a novel structure conjugated with a malonyl-sugar and uronic acid moiety, hitherto an unreported combination. Such structural modifications of blumenols could be significant for their previously reported functional roles associated with the establishment and maintenance of AM colonisation. Pyrrolizidine alkaloids (PAs), key anti-herbivore defence compounds in ragwort, dominated the metabolomic profiles of root and shoot extracts. Analyses of the metabolomic profiles revealed an increase in four PAs in roots (but not shoots) of AMF colonised plants, with the potential to protect colonised plants from below-ground organisms.

**Electronic supplementary material:**

The online version of this article (10.1007/s10886-017-0921-1) contains supplementary material, which is available to authorized users.

## Introduction

Plant-mediated interactions between organisms above- and below-ground are increasingly recognised as a structuring force in ecology, though in many systems there is still a need for a more mechanistic understanding of the chemical basis of these effects (Johnson et al*.* 2012, 2016; Wardle et al*.* 2004). Microbial symbionts have significant impacts on the chemical composition of their host plant, so are potentially key drivers of these indirect interactions (Hartley and Gange 2009). One of the most ancient and widely occurring plant-symbiont relationships involves arbuscular mycorrhizal fungi (AMF) (Smith and Read 2008). AMF are obligate symbionts that, via specialised structures (arbuscules) within the roots of their hosts, enhance plant uptake of macronutrients, particularly phosphorus and nitrogen (Hodge 2016; Hodge and Storer 2015; Javot et al. 2007; Leigh et al. 2009; Thirkell et al. 2016). In return, AMF are wholly dependent on the host plant for their carbon provision, usually acquiring around 10% of host photosynthate (Bago et al. 2000). These nutrient and carbon exchanges modify the nutrient status of the host plant, but also affect the allocation to secondary metabolites and other resistance mechanisms (Cameron et al. 2013; Minton et al*.* 2016). For example, colonisation by AMF has been shown to increase plant resistance to below-ground antagonists such as parasitic plants (Li et al. 2012) and plant parasitic nematodes (Rodriguez-Echeverria et al. 2009), as well as to generalist below-ground herbivores (Vannette and Rasmann 2012). Plant interactions with above-ground herbivores can also be affected by AMF colonisation (van der Putten et al. 2001), although usually to a lesser degree than below-ground (Van Wees et al. 2008), and outcomes vary with AMF species identity, herbivore diet breadth and/or feeding mode (Koricheva et al. 2009).

Early studies examining the impact of AMF colonisation on the chemical protection of plant tissues usually targeted specific chemical groups, limiting their power to detect the full range of induced chemical responses to AMF presence (de Deyn et al. 2009; Eftekhari et al. 2012). More recently, non-targeted metabolomic approaches have been used to investigate the effects of AMF on a wide range of metabolites (the metabolome) in foliar tissues (Fester et al. 2011; Schweiger et al. 2014; Schweiger and Muller 2015). Again, several of these did not measure effects on secondary metabolites, so much remains to be discovered about the potential mechanisms by which AMF colonisation of plant roots can affect above-ground plant-herbivore interactions (Gange et al. 2012; Schweiger and Muller 2015). Similarly, the impact of AMF colonisation on the root metabolome has been little studied, although Schliemann et al. (2008a) used a metabolomic approach to show that both primary and secondary metabolites differed between the roots of *Medicago truncatula* L. individuals with and without AMF colonisation, and Saia et al. (2015) studied impacts of colonisation on the amino acid content of wheat. However, neither of these studies examined above-ground foliar impacts, whilst none of the foliar studies (Fester et al. 2011; Schweiger et al*.* 2014; Schweiger and Muller 2015) examined root tissue. Therefore, hitherto there has been no metabolomic investigation into the concomitant above- and below-ground changes in plant secondary chemistry following AMF colonisation, an important omission given the growing interest in above-belowground links and systemic defence responses (Johnson et al. 2016).

This study addresses this knowledge gap by using non-targeted metabolomics profiling techniques to examine the simultaneous effects of arbuscular mycorrhizal (AM) colonisation on plant secondary chemistry in foliar and root tissues of ragwort (*Senecio jacobaea* L.), a species which forms symbiotic associations with AMF (Gange et al. 2002; Reidinger et al*.* 2012). We used experimental and untargeted metabolomic approaches to test the effect of AMF (*Rhizophagus irregularis*) colonisation on the secondary chemistry of ragwort roots and shoots. We aimed to identify the chemicals induced or repressed in host root and shoot tissues as a result of AM colonisation. Effects of colonisation on host metabolites might be expected to differ between the two tissue types given the established differences in local vs systemic responses to AMF (Van Wees et al. 2008), and, whilst recent research suggests high specificity in leaf metabolomic responses to AMF (Schweiger et al. 2014), this is as yet untested in roots. Hence, we address two questions:

1. How does colonisation by AMF affect the profiles of secondary metabolites in ragwort and are the impacts more pronounced in root vs. foliar tissue?
2. Are the metabolites induced by AM colonisation similar to those identified in other plant-fungal systems, or is there evidence of novel compounds, potentially indicating a degree of host specificity?

## Materials and Methods

### Microcosm Experiment

Ragwort plants were grown from seeds collected from a single plant on the University of Sussex campus (Lat: 50°52′18.6366″N, Long: 000°04′59.6860″W), sterilised in a 5% bleach solution and germinated on double autoclaved vermiculite. Thirty-one pots (1.7 L) received a double autoclaved (121 °C; 60 min) growth medium comprising a 1:1 mixture of silver sand:Terragreen® (an attapulgite clay; Turf-Pro Ltd., UK) and bone meal (0.25 g/L^−1^), a complex N and P source to encourage mycorrhizal development (Hodge and Fitter 2010). After mixing the growth media, all pots were twice flushed to saturation with water, aiming to remove the pulse of nutrients released following sterilisation (Troelstra et al. 2001). Seedlings were assigned to replicate pots and randomly assigned to AMF (*n* = 16) and control (*n* = 15) treatments. Each replicate in the AMF treatment was inoculated with 50 g (dry weight) of granular *Rhizophagus irregularis* (see Redecker et al. 2013 for recent changes in nomenclature; obtained from PlantWorks, Kent, UK). Those replicates assigned to the control treatment also had 50 g of this granular inoculate added, but double autoclaved (121 °C; 60 min) to kill the *R. irregularis*, to control for the input of organic material with the inoculation (see Hodge 2001). In addition, non-AM pots received 100 ml of filtered washings of the *R. irregularis* inoculum, but with AMF propagules removed to limit initial differences among pots in starter microbial communities (as Atkin et al. 2009). To account for any differences caused by the addition of water, 100 ml of deionised water was applied to the AMF-treated microcosms. Each microcosm received a weekly dose of 50 ml of half-strength Rorison’s solution (Hewitt 1966), but with the plant-available phosphate removed to encourage AMF colonisation. The experiment ran for 10 weeks in a greenhouse maintained at 15–25 °C with supplementary lighting (400 W, high pressure sodium lamps) on a 16:8 L:D photoperiod. Microcosms were watered with tap water *ad libitum*.

Plants were destructively harvested after 10 weeks in treatment. From each replicate three new leaves (fully mature closest to the centre of the rosette) and two old leaves (closest to the edge of the rosette with no visible signs of senescence) were excised, weighed, immediately snap frozen in liquid nitrogen to halt enzymatic processes and stored in a − 80 °C freezer for metabolomic analysis. Two representative root samples (each ~10% of the total root mass) per plant were weighed and used for: i) plant metabolomic analysis (snap frozen then stored at −80 °C) and ii) to measure AMF colonisation (stored at 4 °C until processed). Root sections were cleared in a 10% potassium hydroxide solution, ink-stained (Vierheilig et al. 1998), slide mounted and the presence or absence of AMF structures was recorded for a minimum of 100 root intersections using a compound microscope (× 200 magnification). The numbers of arbuscules, vesicles and root length colonized (RLC; the percentage of total intercepts where hyphae or other AM fungal structures were present) were recorded for each intersection (as Hodge 2003). The remaining shoot and root material was weighed and then oven-dried (60 °C, 72 hr) to obtain dry biomass.

### Plant Extraction and Non-targeted Metabolomic Profiling

A homogenised sub-sample (0.1 ± 0.01 g), of the root and shoot tissue taken from each replicate was mixed with 2 mL of a 3:3:2 solvent mix of isopropanol, acetonitrile and water using a ball mill (Pulverisette 23. Fritsch, Germany). This solvent mixture extracts both polar metabolites (carbohydrates, amino acids) and non-polar metabolites (lipids) (Sana et al. 2010). Samples were spiked with two deuterated internal standards; 17β-estradiol 2,4,16,16-*d*_4_ sodium 3-sulfate (E2-*d*_4_-S, >99% D atom) and progesterone-2,2,4,6,6,17α,21,21,21-*d*_9_ (P-*d*_9_, 98% D atom) (Cambridge Isotope Laboratories Inc. MA and CDN isotopes, Quebec, Canada, respectively). The sample was vortexed (1 min) and after overnight extraction at −20 °C, samples were centrifuged, the supernatant removed and the pellet extracted with 2 mL of 80% methanol for 12 hr. The supernatants were combined and a 3 mL aliquot was evaporated to dryness under vacuum and redissolved in 160 μl of methanol:water (3:1, *v*/v). The extract was filtered (0.2 μm) prior to MS analysis. All extraction chemicals used were purchased from Rathburn Chemicals Ltd., Walkerburn, UK.

Metabolites were profiled using ultraperformance liquid chromatography quadrupole time-of-flight mass spectrometry (UPLC-QTOFMS) (Waters, Manchester, UK). Aliquots of plant extracts were injected on to an Acquity UPLC BEH C18 column (1.7 μm particle size, 2.1 × 100 mm, Waters, UK). Metabolites were separated using a water-formic acid and acetonitrile gradient as follows: 0–9.0 min, from 0 to 30.0% acetonitrile; 9.0–15.0 min, from 30.0 to 100% acetonitrile; 15.0 to 23.0 min, 100% acetonitrile. The flow rate was 0.2 mL min^−1^ and the column temperature was 30 °C. The injection volume of plant extracts was 20 μl.

Metabolites were detected in both positive and negative ESI modes using a Micromass TOF-MS system (Waters, Manchester, UK). The mass spectrometer was tuned to 9000 mass resolution and data collected in full scan mode from 100 to 1200 *m/z*. The collision gas used was argon, a constant collision energy of 10 eV was used for all experiments, and the TOF penning pressures ranged from 4.63 × 10^−7^ to 4.83 × 10^−7^ mbar. Capillary voltage was 2.6 in positive mode and −2.9 in negative mode. In positive mode, the cone voltage was set at 36 V, and the multiplier voltage was set at 654 V. In negative mode the cone and multiplier voltage were set at 35 V and 550 V respectively. Desolvation N_2_ gas flow was set at 401 L h^−1^ for both ionisation modes.

MarkerLynx software (V 4.1, Waters, Manchester, UK) was used to align, normalise and remove isotopic peaks from the metabolomic profiles. Each metabolite signal was the description of an analyte using its specific retention time (r.t.) and mass-to-charge ratio (*m/z*). Using SIMCA-P multivariate analysis software (Umetrics UK Ltd., Windsor, UK) the data were pareto scaled, log-transformed and modelled using partial least square-discriminate analysis (PLS-DA) for >2 classes, or orthogonal partial least-square discriminate analysis (OPLS-DA) for comparison of just 2 classes followed by examination of the loading plots to detect MS signals associated with AMF colonisation (Liland 2011; Trygg and Wold 2002). For all multivariate analyses, the explained variation (R^2^Y) and the predictive power of the model (Q^2^) were examined to assess the performance of the models.

Biochemical markers associated with AM colonisation were extracted from ‘S’-plots derived from the OPLS-DA models (Wiklund et al. 2008). The identity of biochemical markers was determined from their accurate mass composition and isotopic fit using the elemental composition tool from the MassLynx software (V 4.1, Waters, Manchester, UK). Fragmentation data, obtained from collision induced dissociation (CID) using QTOFMS (collision energy between 20 and 50 eV) was used to confirm the putative identity of the markers.

### Analyses of Pyrrolizidine Alkaloids in Metabolomic Profiles of Tissue Extracts

Metabolomic profiles of root extracts acquired in positive electrospray ionization (ESI) mode were dominated by highly abundant pyrrolizidine alkaloids (PAs) saturating the detector response. PAs are the principal secondary metabolites in *Senecio* species, synthesised in the roots (Hartmann et al. 1989) before being transported to the shoots (Hartmann and Dierich 1998) where they are effective defences against generalist herbivores (Macel et al. 2005; Narberhaus et al. 2005; Thoden et al. 2009). In order to determine whether PA profiles changed as a result of AM colonisation, an additional analysis of a 0.5 μl injection of root extracts was undertaken to reduce the highly abundant PA signals. The chromatograms (of both root and shoot extracts) were manually searched, and the relative abundance of observed signals corresponding to PA metabolites was determined relative to the internal standard using MassLynx peak integration software. PAs were assigned putative identities as described above. Where possible, authenticated standards were purchased to confirm PA identity: a mixed standard of PAs seneciphylline and senecionine (Carl Roth, GmbH & Co, Karlsruhe, Germany) and a retrorsine N-oxide standard (PhytoLab GmbH & Co. KG, Nürnberg, Germany). Details of the MS fragmentation data used for the identification of each metabolite are outlined in the supporting information.

### Data Analysis

Adjusted fresh weights ((total fresh weight/100)*water content) of plant tissues were calculated to account for a significant between-treatment difference in root and shoot water content (water content = (fresh weight-dry weight)/(fresh weight)*100). Statistical analyses of differences in plant biomass, biochemical markers and AM colonisation between treatments were performed in SPSS v11. Where the residuals of the data met the assumptions, either before or following transformations, of parametric models (normality, homogeneity of variance), then one-way ANOVA (for plant biomass) or t-tests (for percentage RLC, metabolite and PA concentrations) were used. For non-parametric data, Mann-Whitney U tests were employed. Bonferroni corrections were used to account for the increased false discovery rate (FDR) associated with the multiple testing of metabolic datasets containing many thousands of metabolite signals (Broadhurst and Kell 2006). A less conservative Benjamini and Hochberg FDR correction was applied to the univariate tests of PA concentrations (Benjamini et al. 2001), as this was more appropriate for these much smaller datasets. The association between blumenol metabolites and percentage RLC were investigated using Spearman rank-order correlations, as the data did not meet the assumptions of parametric tests.

## Results

### AMF Colonisation and Plant Biomass

Mean percent RLC was 47 ± 4% in the *R. irregularis* colonised plants. Frequency of arbuscules was 16 ± 1% and vesicles 13 ± 2% respectively. There was no AMF colonisation of the non-AM controls. Shoot and root water content were higher in control plants than in *R. irregularis* colonised plants, though plant biomass was unaffected by *R. irregularis* colonisation (Table S1).

### Root metabolome

**Fig. 1:**
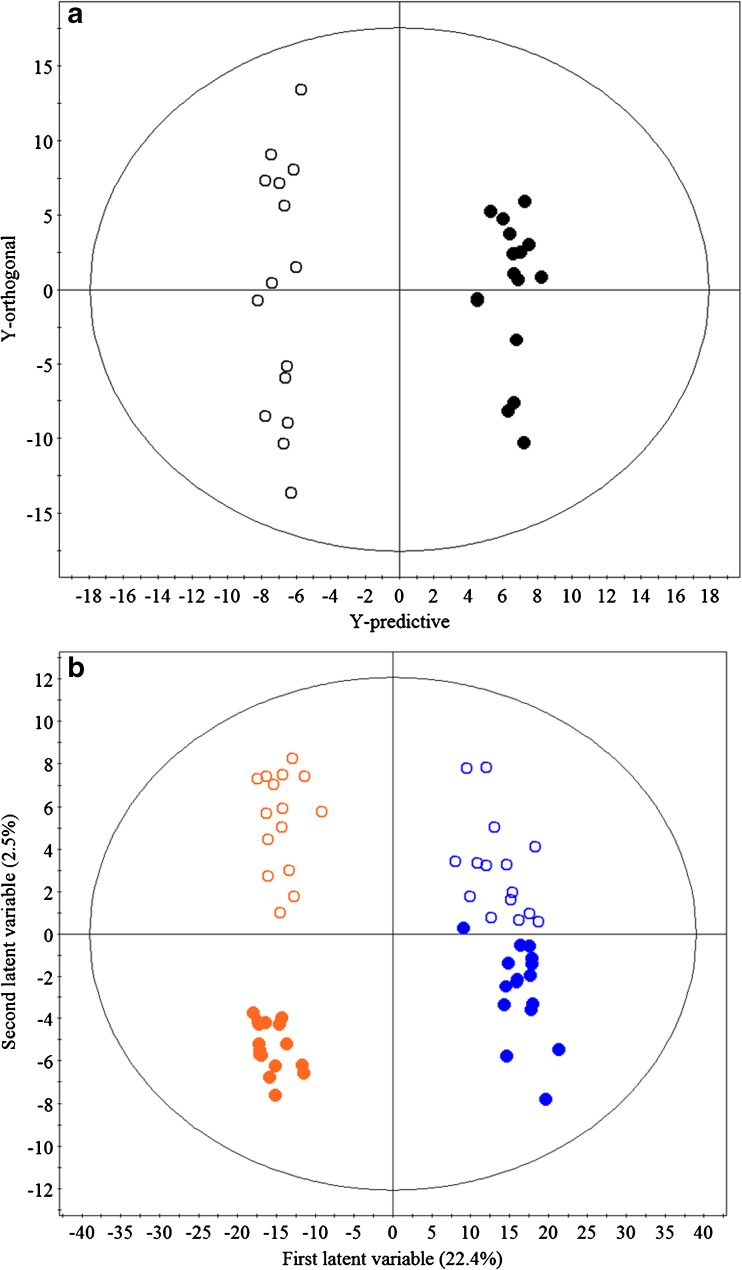
**a** Orthogonal partial least square-discriminate analysis (OPLS-DA) scores plot of the chemical profiles of root extracts from control and *Rhizophagus irregularis* treated ragwort, **b** Partial least square-discriminate analysis (PLS-DA) scores plot of the chemical profiles of leaves from control and *R. irregularis* treated ragwort. Open and closed circles represent control and *R. irregularis* treated ragwort plants, respectively. In **b**, blue symbols represent samples from new leaves and orange symbols those from old leaves and the percentages of explained variation (R^2^Y) modelled by the first two latent variables are displayed on the axes. Both plots show datasets from samples profiled in positive ESI MS mode; data from negative ESI mode revealed similar model characteristics (Fig. S1)

The metabolite profiles of root extracts from AM colonised and non-AM control plants were clearly separated, indicating a shift in chemical composition of plants in the different treatment groups; (see Fig. 1a for the positive ESI mode dataset, and a similar scores plot was obtained for the negative ESI mode in Fig. S1a). OPLS-DA of both datasets (positive and negative ESI modes) produced models that explained a large amount of the variation in metabolite profiles between treatments (negative: R^2^Y = 0.99, positive: R^2^Y = 0.98) with good predictability (negative: Q^2^ = 0.53, positive: Q^2^ = 0.48). This separation between treatment classes was driven by increased concentrations of 33 metabolites associated with AM colonisation (threshold values after Bonferroni adjustment: positive mode: *P* < 5.20 × 10^−7^; negative mode: *P* < 5.01 × 10^−7^). The fold increase in concentration of the 33 metabolites associated with AM roots was between two to many thousands (up to 37,995-fold in one instance). Eight of these metabolites were assigned identities (Table 1); the remaining metabolites (*n* = 25) are listed in Table S2. Blumenol standards are not commercially available, so identifications are derived from comparisons with published mass spectra (cited in Strack and Fester 2006; Peipp et al. 1997; Schliemann et al. 2008b); see supporting information (Note 1, Fig. S2 and Table S3) for further details.

**Table 1:** Metabolites identified in ragwort roots that were significantly increased after colonisation by *Rhizophagus irregularis*

Metabolite number^¶^	Observed ion (*m/z*)	UPLC- TOFMS r.t.	Putative formula	Theoretical mass of ion	Putative identity	Fold change ^a^	*P*-value ^b^
1	389.2177	6.37	C_19_H_33_O_8_	389.2175	13-hydroxyblumenol C glycoside [M + H]^+^	1151.2*	6.65 × 10^−9^
1	387.2018	6.37	C_19_H_31_O_8_	387.2018	13-hydroxyblumenol C glycoside [M-H]^−^	746.6*	6.65 × 10^−9^
2	373.2228	7.23	C_19_H_33_O_7_	373.2226	Blumenol C glycoside [M + H]^+^	8065.8	6.65 × 10^−9^
3	547.2389	7.30	C_25_H_39_O_13_	547.2391	Blumenol C glycosyl-glucuronide [M-H]^−^	1553.3	6.65 × 10^−9^
4	475.2182	7.55	C_22_H_35_O_11_	475.2179	13-hydroxyblumenol C malonylglycoside [M + H]^+^	489.7	2.99 × 10^−7^
5	373.2222	8.20	C_19_H_33_O_7_	373.2226	Blumenol C glycoside [M + H]^+^	5032.0	6.65 × 10^−9^
6	635.2551	8.24	C_28_H_43_O_16_	635.2551	Blumenol C malonylglycosyl-glucuronide [M + H]^+^	3101.3*	6.65 × 10^−9^
6	633.2396	8.24	C_28_H_41_O_16_	633.2395	Blumenol C malonylglycosyl-glucuronide [M-H]^−^	250.4*	6.65 × 10^−9^
7	459.2232	9.38	C_22_H_35_O_10_	459.2230	Blumenol C malonylglycoside [M + H]^+^	1109.2	6.65 × 10^−9^
8	494.3247	14.20	C_24_H_49_NO_7_P	494.3247	Hexadecenoyl-glycero-phosphocholine [M + H]^+^	7.86	2.00 × 10^–7c^

^¶^Structures given in Fig. 2

r.t. = retention time

^a^Fold change indicates the concentration increase in roots colonised with AMF when compared to the concentrations observed in control plants

^b^Significance determined using t-tests (^c^) or Mann-Whitney U tests (unmarked) after Bonferroni adjustments

* Differences in estimation of fold change between [M + H]^+^ and [M-H]^−^ signals for the same metabolite were due to presence of a Na adduct competing with the [M + H]^+^ ion in positive ESI mode

**Fig. 2:**
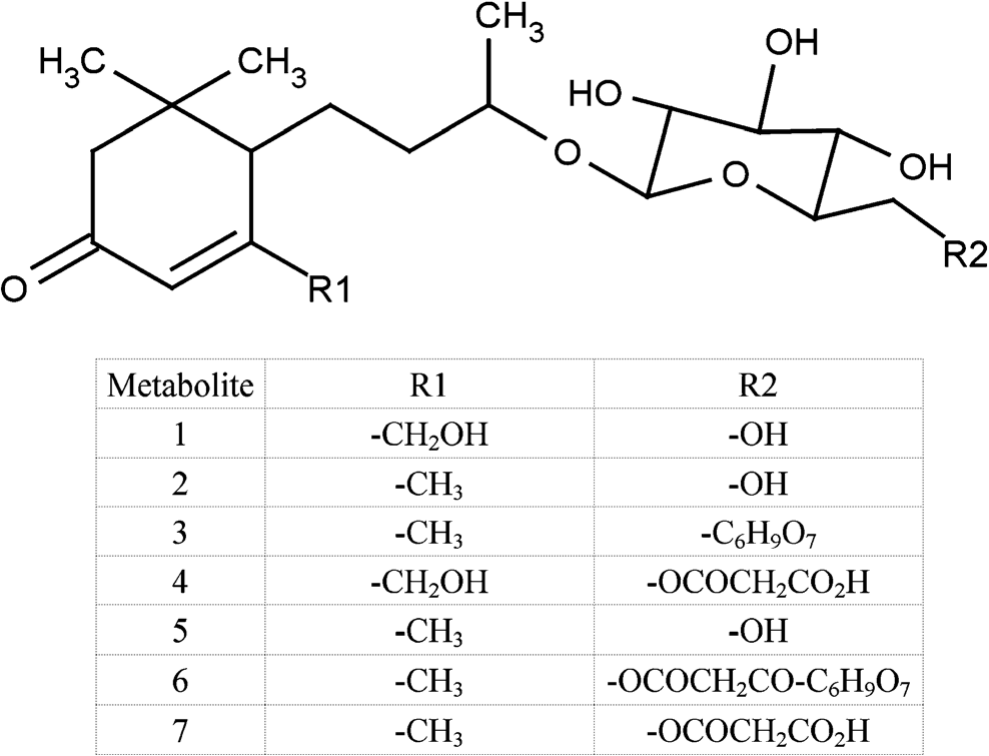
Schematic structure of the identified blumenol apocarotenoids associated with *Rhizophagus irregularis* colonisation of ragwort roots

Seven of the identified metabolite signals were conjugates of C_13_ cyclohexenones, i.e. blumenol apocarotenoids (metabolites 1-7, Table 1, Fig. 2). Metabolites 1 and 4 were identified as blumenols with a 13-hydroxyblumenol C moiety (C_13_H_23_O_3_). Metabolites 2, 3, 5, 6 and 7 were identified as blumenols with a blumenol C moiety (C_13_H_23_O_2_). Whilst identified metabolites 1–7 share common blumenol structures, they differed in the nature of conjugation (Fig. 2). Metabolites 1, 2 and 5 were conjugated with glycoside sugars, whereas metabolites 4 and 7 were conjugated with malonylglycoside sugars. Metabolites 3 and 6 were conjugated with an uronide group consistent with a glucuronide (Strack and Fester 2006), and so were identified as a glycosyl-glucuronide and a malonylglycoside-glucuronide conjugate respectively. Metabolite 6, blumenol C malonylglycosyl-glucuronide, is a hitherto unreported structure, whereas metabolites 1–5 and 7 have been found to be associated with AM colonisation in other species (Peipp et al. 1997; Schliemann et al*.* 2008b). All blumenols were present as their molecular ions, which were either [M + H]^+^ or [M-H]^−^ in positive or negative ESI, respectively. In positive ESI, some metabolites were also present as the sodium adduct of the molecular ion (Table S3). Of those blumenols identified, two were observed in both positive and negative ESI modes (metabolites 1 & 6, Table 1). The concentration changes of the identified blumenols corresponded to large differences, with fold increases ranging from 250 to over 8000 in AM plants. The final identified metabolite (number 8) was determined to be hexadecenoyl-glycero-phosphocholine, a lysophospholipid.

All blumenol apocarotenoids were positively associated with increasing percentage RLC in the AM colonised plants (Spearman correlation coefficient 0.523–0.711, *P* ≤ 0.03–0.002, Table 2). Levels of hexadecenoyl-glycero-phosphocholine were not significantly correlated with RLC (*P* ≥ 0.11). A number of unidentified metabolites were detected that were associated with AM colonisation (Table S2) and these were likely to be conjugated phenolic compounds, two additional conjugated blumenols and (in one case) a phospholipid. Based on their empirical formula, none of the metabolites (except for the phospholipid) were present in the plant metabolite databases and so potentially represent new structures. However, their concentrations were too low to allow further structural characterisation. Changes in the concentrations of primary metabolites were not observed as a result of AM colonisation, but this was most likely due to the fact that some structures such as lipids and carotenoids were not have been detected by ESI and small organic molecules, including alkaloid precursors, would not have been retained on the HPLC column.

**Table 2:** Spearman rank-order correlations (*N* = 16) between the concentration of the blumenol metabolites measured in ragwort roots and *Rhizophagus irregularis* colonisation in the AMF-treated plants

Metabolite structure (see Fig. 2)	Putative identity	Correlation coefficient	*P*-value^¶^
1	13-hydroxyblumenol C glycoside	0.852	1.16 × 10^−9^
2	Blumenol C glycoside	0.902	4.33 × 10^−12^
3*	Blumenol C glycosyl-glucuronide	0.828	9.37 × 10^−9^
4	13-hydroxyblumenol C malonylglycoside	0.841	3.21 × 10^−9^
5	Blumenol C glycoside	0.844	2.41 × 10^−9^
6	Blumenol C malonylglycosyl-glucuronide	0.832	6.39 × 10^−9^
7	Blumenol C malonylglycoside	0.855	9.36 × 10^−10^

*Result from MS analysis in -ESI mode (required to detect metabolite 3); all other results from +ESI mode

^¶^All *P*-values significant after Bonferroni adjustment

### Shoot Metabolome

PLS-DA models of the shoot dataset from positive ESI analysis revealed good predictability (Q^2^ = 0.66) and a high degree of explained variation (total R^2^Y = 0.99 for six latent variables) (Fig. 1b). PLS-DA recorded in negative ESI mode revealed similar models that explained a high level of variation in shoot chemistry (R^2^Y = 0.62, 4 latent variables) with lower predictability (Q^2^ = 0.36) than in positive mode (Fig. S1b). In both positive and negative modes, the first latent variable separated the metabolomes of old and new leaves. The second, whilst separating AM and non-AM control groups, only explained a relatively small amount of the observed variation in metabolite profiles between these treatments. OPLS-DA models with the highest predictability (R^2^Y = 0.989, Q^2^ = 0.431) were associated with the positive ESI profiles of old leaves from the different treatment groups. However, in all OPLS-DA models of shoot tissues, no metabolites were found to vary significantly in association with AMF colonisation of ragwort.

### Concentrations of PA Metabolites in the Root and Shoot Metabolome

Analysis of the UPLC-QTOFMS chromatograms from the metabolomics study revealed that signals of PA metabolites were saturated in samples of the root extracts. Analysis of lower volumes of samples revealed 19 PA signals that could be resolved in the metabolomic profiles of roots, however more PA structures (potentially up to 33 metabolites, Kostenko et al. 2013) were likely to be present, which were not detected due to masking of their signals by the most abundant PA signals. The concentration of individual PAs within a species or genotype varies greatly in both roots and shoots (Cheng et al. 2011ba). Of the detected PAs, four were significantly increased in AM plants (Table 3). The PAs that responded to AMF colonisation were identified as senecionine, jacoline N-oxide, jaconine N-oxide, and usaramine N-oxide. Of these four PAs, only levels of senecionine were significantly correlated with RLC (Spearman correlation coefficient − 0.560, *P* ≤ 0.02). Details of the identification of the different PA structures are given in supporting information.

**Table 3:** Average relative concentrations (±S.E.) of the pyrrolizidine alkaloid (PA) signals measured in the positive ESI UPLC-TOFMS profiles of control (*N* = 15) and *Rhizophagus irregularis* colonised (*N* = 16) ragwort roots

Theoretical mass of ion	Putative formula	UPLC-TOFMS r.t.	Control	*G. intraradices* colonised	*P*-value^a^	Fold change^b,c^	Putative identity^c^
334.1654	C_18_H_23_NO_5_	6.00	49.7 (±5.3)	59.2 (±5.3)	0.188		
336.1811	C_18_H_25_NO_5_	6.86	211.7 (±17.7)	289.8 (±21.6)	3.63 × 10^−3^	1.37	Senecionine
350.1604	C_18_H_23_NO_6_	6.34	220.5 (±27.7)	223.2 (±21.6)	0.571		
	C_18_H_23_NO_6_	9.01	24.5 (±3.2)	38.6 (±6.2)	0.054		
352.1760	C_18_H_25_NO_6_	4.89	23.7 (±8.7)	52.0 (±14.2)	0.033		
	C_18_H_25_NO_6_	5.82	76.1 (±11.3)	55.3 (±5.4)	0.216		
	C_18_H_25_NO_6_	6.13	327.9 (±23.9)	303.8 (±17.9)	0.711		
	C_18_H_25_NO_6_	6.99	48.8 (±2.5)	53.0 (±2.4)	0.029		
	C_18_H_25_NO_6_	7.16	108.8 (±4.2)	113.0 (±3.5)	0.110		
366.1553	C_18_H_23_NO_7_	4.29	193.2 (±46.1)	174.4 (±31.4)	0.598		
	C_18_H_23_NO_7_	4.98	126.0 (±33.8)	141.9 (±39.5)	0.891		
368.1709	C_18_H_25_NO_7_	5.24	45.9 (±6.7)	65.2 (±10.2)	4.81 × 10^−3^	1.42	Usaramine N-oxide
	C_18_H_25_NO_7_	5.62	93.8 (±20.2)	119.3 (±18.8)	0.231		
	C_18_H_25_NO_7_	6.39	550.5 (±54.4)	530.8 (±38.6)	0.953		
370.1866	C_18_H_27_NO_7_	5.94	24.0 (±2.2)	25.8 (±2.8)	0.379		
376.1760	C_20_H_25_NO_6_	9.55	118.5 (±12.1)	118.4 (±14.3)	1.000		
386.1815	C_18_H_27_NO_8_	3.68	34.6 (±9.9)	105.6 (±16.0)	2.01 × 10^−3^	3.05	Jacoline N-oxide
392.1709	C_20_H_25_NO_7_	9.77	196.5 (±26.17)	194.0 (±31.4	0.740		
404.1476	C_18_H_26_NO_7_Cl	5.22	39.3 (±9.73)	122.2 (±13.5)	5.87 × 10^−5^	3.11	Jaconine N-oxide

Relative concentrations were quantified as the ratio of the analyte signal: internal standard signal per 0.3 mg plant mass

^a^Results that were significant after Benjamini and Hochberg corrections (Benjamini et al. 2001) are displayed in bold

^b^Fold change indicates the concentration increase in roots colonised with AMF when compared to the concentrations observed in control plants

^c^Only given for those PAs that differed significantly between treatment groups

The relative abundance of PAs was also determined in the metabolomics datasets of the shoot extracts. A total of 17 abundant PA structures were resolved in the metabolomics profiles of the shoot extracts out of a potential of 29 PA metabolites (Kostenko et al. 2013). However, there were no significant differences in concentrations (after FDR corrections) of PA signals between control and AMF-colonised treatment groups which was consistent with the lack of discriminating metabolites observed in the above study on the shoot metabolome (Table S4).

## Discussion

This study, the first to use a non-targeted metabolomic approach to assess the effect of AMF colonisation on host plant secondary chemistry in both roots and shoots, showed that colonisation by AMF could increase the concentrations of 33 metabolites in roots by between two and many thousand fold. The extent of AMF colonisation was positively correlated with levels of compounds thought to be associated with establishing and maintaining AMF colonisation, specifically seven C_13_ cyclohexenone blumenol structures, including one with a hitherto unidentified structure. In addition, AMF colonisation affected the levels of the main anti-herbivore defences in root tissues, with four PAs significantly increased in concentration. Despite these below-ground changes, no significant changes in the metabolome of above-ground tissues were detected in response to AMF colonisation of roots, although there were clear metabolomic differences between leaf tissues of different ages. This is noteworthy as had changes been found in aboveground tissues then it would indicate the AM fungus had a direct impact upon the plant. However, as differences were only found in the ‘mycorrhizal’ (literally, ‘fungus-root’) root tissue, an unknown fraction of the metabolomics response observed here, and in other studies (e.g. Rivero et al. 2015; Saia et al. 2015), may be due directly to the fungal partner. Experimentally distinguishing between responses driven by the AMF, the root and the mycorrhizal root is hampered by the fact that many of these fungal symbionts cannot be grown in the absence of a host plant (Hodge et al. 2010). Nevertheless, our results demonstrate that colonisation of roots by AMF results in clear metabolomics differences compared to the un-colonised condition, a significant finding given that c. two-thirds of land plants form this type of close symbiotic association (Hodge and Fitter 2013; Smith and Read 2008).

The process of initiating and maintaining the plant-AMF relationship involves a range of metabolic changes within the host (Fester et al. 2011; Schliemann et al. 2008a), and the bumenol metabolites we detected in our non-targeted analysis are of particular significance, as they are thought to be involved in the chemical signalling underpinning the plant-AMF interaction (Walter et al. 2010; Maier et al. 1995). It has been suggested that blumenols and C14 polyene apocarotenoids have a role in maintaining the plant-AMF interaction once formed (Fester et al. 2002). For instance, concentrations of root C14 polyenes and blumenols increase (Peipp et al. 1997; Schliemann et al. 2006, 2008b) as the plant-AMF interaction progresses, particularly in root cells hosting AMF structures (Fester et al. 2002). Mutant plants, where blumenol production is reduced, are associated with an increased amount of dead and degenerating mycorrhizal structures (Floss et al. 2008), providing further evidence that these blumenol C13 cyclohexenones may have a role in the persistence of AMF colonisation (Walter et al. 2010).

In support of this hypothesis, and in accordance with other studies (Maier et al. 2000; Schliemann et al. 2008a), the concentrations of the seven blumenols we identified were all positively related to levels of AMF colonisation. One of these induced blumenols (metabolite 6) was conjugated with a malonyl-sugar and glucuronic acid moiety, a combination that has never been reported before. Species-specific blumenol metabolites have been observed in a range of plant taxa (Fester et al. 2002, 2005; Maier et al. 2000; Peipp et al. 1997; Schliemann et al. 2006, 2008a), and have been suggested as a mechanism of species recognition between the AMF and its host (Strack and Fester 2006). This is a hypothesis we raise for further testing, but current data is sparse. So far, only a few other plant species, such as the legume *Medicago truncatula* colonised by *Glomus intraradices* (Schliemann et al. 2008a), tomato colonised by *R. irregularis* and *Funneliformis mosseae* (Rivero et al. 2015) and wheat colonised by multiple AM species (Saia et al. 2015) have had the root tissue response to AMF colonisation been examined in an untargeted metabolomic approach. Until a wider variety of plant-AMF species combinations are tested, it is too early to conclude whether any species-specific signalling exists in AMF-plant interactions. It is notable, however, that one recent study (Schweiger et al. 2014) which compared the impacts of the AMF *Rhizophagus irregularis* on the leaf metabolome of five different plant species found that species-specific metabolic changes following colonisation far out-numbered more generic cross-species responses. Similarly, Rivero et al. (2015) detected fungus-specific aspects of the AM-associated changes in the tomato metabolome.

Despite substantial changes to the root metabolome induced by AMF colonisation, there were no concomitant shifts in the aboveground metabolome. Conceivably, changes in the shoot metabolome may have been too small or inconsistent to have been detected, but this seems unlikely given that our UPLC-QTOFMS methodology clearly discriminated between the metabolomes of old and new leaves (Fig. 1b), and that we have used the same technique to successfully detect differences in the metabolomes of above-ground tissues in ragwort previously (Hartley et al. 2012). Previous studies have demonstrated that AMF-mediated changes occur in above-ground plant tissues, with effects on gene expression (Taylor and Harrier 2003) and on herbivore performance (Koricheva et al. 2009), but changes in foliar secondary metabolites in response to colonisation seem more inconsistent. It has been suggested that induction of chemical defences by beneficial microbes are localised rather than systemic (Van Wees et al. 2008), which may explain why the increases in PA concentration in AMF colonised ragwort we observed were confined to the roots. Increases in PA concentrations in the roots may even reduce PA levels in the shoots. Although our study did not detect such a reduction in foliar allocation to PAs, Reidinger et al. (2012) showed that leaf concentrations of both total PAs and jacoline were negatively correlated with AMF colonisation of ragwort in the field.

The increased PA levels that we found in AMF colonised roots could impact on trophic interactions. AMF colonisation has been shown to have a negative influence on generalist below-ground herbivores such as parasitic nematodes (Li et al. 2006; Rodriguez-Echeverria et al. 2009) and beetle larvae (Gange et al. 1994), but positive effects on the root-feeding larvae of specialist weevils (Currie et al. 2011). Overall, generalist herbivores, whether above or below ground, seem more affected by AMF colonisation of their host plants than specialists (Gange et al. 2012; Koricheva et al. 2009). In ragwort, the highly toxic PAs deter generalist herbivores (Joosten and van Veen 2011; Thoden et al. 2009), whilst specialist herbivores have the capacity to use PAs to locate and select hosts (Cheng et al. 2013), to detoxify them, and even sequester these compounds for their own defence against predators (Beuerle et al. 2007; Narberhaus et al. 2003). Most previous studies have demonstrated these effects on above-ground herbivores (e.g. Cheng et al. 2011b; Wei et al. 2015); far less is known about the impacts of PAs on root-feeding herbivores (Cheng et al. 2011a) and we have found no published studies on how AMF colonisation modifies those effects. It has been shown that root damage increases PA levels (Hol et al. 2004) and that PAs affect root fungi and the composition of the rhizosphere (Hol and van Veen 2002; Kowalchuk et al. 2006), so there is potential for significant ecological consequences deriving from the changes in PAs we observed in AM roots.

In summary, this study has shown that colonisation by the AMF species *R. irregularis* causes numerous and significant changes in the root metabolome of ragwort, but not in the shoot metabolome. In root tissues, the key changes were in groups of chemicals associated with AMF-plant signalling (blumenols) and anti-herbivore defence (PAs). We showed strong correlations between the levels of blumenols and AMF colonisation, supporting the idea that these metabolites play a key role in the interactions between AMF and their hosts. The discovery that one of these was a novel structure not previously reported raises questions about the significance of structural modifications in species recognition and signalling that would repay further study.

## Electronic supplementary material

ESM 1 (DOCX 217 kb)
