# Supplementary material for: Arbuscular Mycorrhizal Fungi and Plant Chemical Defence: Effects of Colonisation on Aboveground and Belowground Metabolomes
Source: J Chem Ecol. 2018 Feb 2;44(2):198–208. doi: 10.1007/s10886-017-0921-1 (PMC5843688; doi:10.1007/s10886-017-0921-1)
Supplement: Supplementary file 1 — (DOCX 217 kb) [file 10886_2017_921_MOESM1_ESM.docx]

SUPPORTING INFORMATION

Table S1 Mean (±S.E.) plant performance parameters (recorded at harvest after 10 weeks growth) of control and *Rhizogphagus irregularis* colonised ragwort.

FIG S1 a) Orthogonal partial least square-discriminate analysis (OPLS-DA) scores plot of the chemical profiles of root extracts from control and *Rhizophagus irregularis* treated ragwort; b) Partial least square-discriminate analysis (PLS-DA) scores plot of the chemical profiles of leaves from control and *Rhizophagus irregularis* colonised ragwort.

Table S2 Unidentified metabolites, observed using UPLC-TOFMS in positive and negative ESI MS modes, that significantly increased in the roots of ragwort colonised with the AMF *Rhizophagus irregularis*.

Note S1 Details the identification process of the metabolites associated with *Rhizophagus irregularis* colonisation of ragwort.

FIG S2 The characteristic fragment patterns obtained as a result of UPLC-QTOFMS CID fragmentation of blumenols.

Table S3 Details of the fragments (obtained using UPLC-QTOFMS CID analyses) used to identify the metabolites that significantly increased in ragwort roots colonised by *Rhizophagus irregularis.*

Table S4 Average relative concentrations (±S.E.) of the pyrrolizidine alkaloid (PA) signals measured in the positive ESI UPLC-TOFMS profiles of control (n=15) and *Rhizophagus irregularis* colonised (n=16) ragwort leaves.

**TABLE 1** MEAN (±S.E.) PLANT PERFORMANCE PARAMETERS (RECORDED AT HARVEST AFTER 10 WEEKS GROWTH) OF CONTROL AND *RHIZOPHAGUS IRREGULARIS* COLONISED RAGWORT

| Plant parameter | Control | With AMF | Test statistic | P-value |
| --- | --- | --- | --- | --- |
| Number of leaves | 16.0 (±0.90) | 18.0 (±1.35) | -0.835^b^ | 0.423 |
| Rosette width (mm) | 246 (±11.95) | 244 (±9.11) | 0.012^a^ | 0.914 |
| Largest leaf length (mm) | 133 (±4.40) | 132 (±2.49) | -0.158^b^ | 0.892 |
| Largest leaf width (mm) | 50 (±2.28) | 53 (±2.50) | 0.866^a^ | 0.360 |
| Largest leaf area (mm) | 6586 (±362.89) | 6955 (±338.12) | 0.555^a^ | 0.462 |
| **Shoot water content (%)** | **88.62 (±0.50)** | **82.37 (±0.54)** | **6.623^a^** | **0.015** |
| **Root water content (%)** | **88.62 (±0.28)** | **87.64 (±0.25)** | **13.032^a^** | **0.001** |
| Shoot weight (g) * | 10.79 (±0.63) | 11.50 (±0.34) | 1.008^a^ | 0.324 |
| Root weight (g) * | 7.76 (±0.69) | 8.31 (±0.52) | 0.412^a^ | 0.526 |
| Total plant weight (g) * | 18.55 (±1.22) | 19.81 (±0.75) | 0.793^a^ | 0.381 |
| Shoot:root ratio | 0.71 (±0.69) | 0.72 (±0.04) | 0.040^a^ | 0.842 |

* Weight data was adjusted prior to analysis to account for a significant difference in root and shoot water content.

^a^ F-ratio (*one-way ANOVA*)

^b^ Z statistic (*Mann-Whitney U tests*)

**
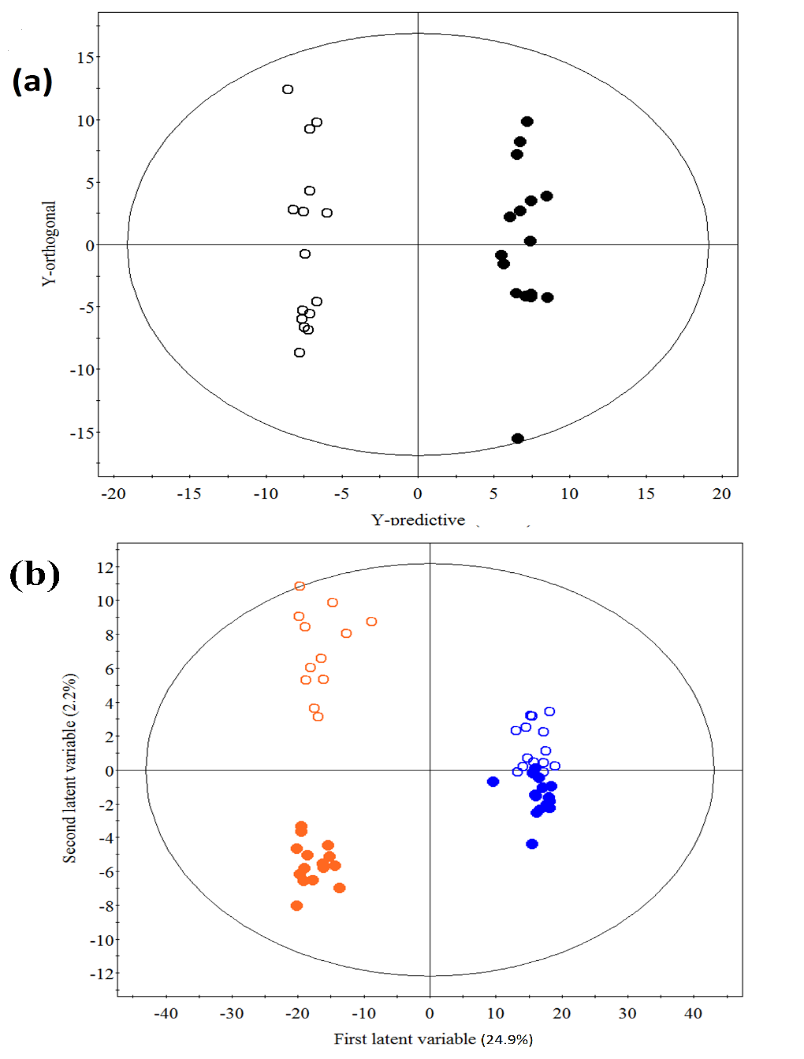
**

FIG S1 A) ORTHOGONAL PARTIAL LEAST SQUARE-DISCRIMINATE ANALYSIS (OPLS-DA) SCORES PLOT OF THE CHEMICAL PROFILES OF ROOT EXTRACTS FROM CONTROL AND *RHIZOPHAGUS IRREGULARIS* TREATED RAGWORT, B) PARTIAL LEAST SQUARE-DISCRIMINATE ANALYSIS (PLS-DA) SCORES PLOT OF THE CHEMICAL PROFILES OF LEAVES FROM CONTROL AND *RHIZOPHAGUS IRREGULARIS* COLONISED RAGWORT

Open circles and closed represent control and *R. irregularis* treated ragwort plants, respectively. In b) blue symbols represent samples from new leaves and orange symbols those from old leaves and the percentages of explained variation (R^2^Y) modelled by the first two latent variables are displayed on the axes. Both plots show datasets for samples profiled in negative ESI MS mode.

TABLE S2 UNIDENTIFIED METABOLITES, OBSERVED USING UPLC-TOFMS IN POSITIVE AND NEGATIVE ESI MS MODES, THAT SIGNIFICANTLY INCREASED IN THE ROOTS OF RAGWORT COLONISED WITH THE AMF *RHIZOPHAGUS IRREGULARIS*

| Ionisation mode | Observed ion (*m/z*) | UPLC-TOFMS r.t. | Putative formula | Theoretical mass of ion | Fold change ^a^ | P-value ^b^ |
| --- | --- | --- | --- | --- | --- | --- |
| Positive | 513.1584 | 7.53 | C_21_H_30_O_13_Na | 513.1584 | 8.73 | 4.42 x 10^-7c^ |
| Positive | 541.2261 | 8.40 | C_24_H_38_O_12_Na | 541.2261 | 31.1 | 4.46 x 10^-7^ |
| Positive | 375.1658 | 9.33 | C_17_H_27_O_9_ | 375.1655 | 11.8 | 6.65 x 10^-9^ |
| Positive | 357.1544 | 9.36 | C_17_H_25_O_8_ | 357.1549 | 5.89 | 4.66 x 10^-8^ |
| Positive | 207.1022 | 9.45 | C_12_H_15_O_3_ | 207.1021 | 2.67 | 2.99 x 10^-7^ |
| Positive | 329.1238 | 9.46 | C_15_H_21_O_8_ | 329.1236 | 8.48 | 1.26 x 10^-7^ |
| Positive | 465.1373 | 9.91 | C_20_H_26_O_11_Na | 465.1373 | 3630 | 2.99 x 10^-7^ |
| Positive | 499.0796 | 9.92 | C_13_H_23_O_20_ | 499.0783 | 5391 | 6.65 x 10^-9^ |
| Positive | 611.1956 | 10.74 | C_26_H_36_O_15_Na | 611.1952 | 13.2 | 3.15 x 10^-11c^ |
| Positive | 645.1353 | 10.74 | C_29_H_31_O_13_NaCl | 645.1351 | 11.7 | 6.65 x 10^-9^ |
| Positive | 985.4650 | 10.99 | C_48_H_73_O_21_ | 985.4644 | 37995 | 6.65 x 10^-9^ |
| Positive | 452.2778 | 14.16 | C_21_H_43_NO_7_P ^d^ | 452.2777 | 10.4 | 2.49 x 10^-7c^ |
| Positive | 462.3026 | 15.43 | C_30_H_40_NO_3_ | 462.3008 | 4.48 | 7.40 x 10^-10c^ |
| Negative | 403.1986 | 5.82 | C_19_H_31_O_9_ ^e^ | 403.1968 | 12.7 | 2.99 x 10^-7^ |
| Negative | 429.2120 | 7.66 | C_21_H_33_O_9_ ^e^ | 429.2125 | 113 | 4.66 x 10^-8^ |
| Negative | 287.1490 | 8.38 | C_14_H_23_O_6_ | 287.1495 | 23.8 | 6.65 x 10^-9^ |
| Negative | 373.1497 | 9.42 | C_17_H_25_O_9_ | 373.1499 | 11.1 | 4.05 x 10^-10c^ |
| Negative | 327.1085 | 9.58 | C_15_H_19_O_8_ | 327.1080 | 4.55 | 1.73 x 10^-9c^ |
| Negative | 677.2062 | 9.58 | C_32_H_37_O_16_ | 677.2082 | 5.37 | 1.33 x 10^-8c^ |
| Negative | 715.2816 | 9.87 | C_33_H_47_O_17_ | 715.2813 | 588 | 6.65 x 10^-9^ |
| Negative | 397.1493 | 10.00 | C_19_H_25_O_9_ | 397.1499 | 339 | 6.65 x 10^-9^ |
| Negative | 927.3853 | 10.00 | C_44_H_63_O_21_ | 927.3862 | 100 | 6.65 x 10^-9^ |
| Negative | 383.1338 | 10.17 | C_18_H_23_O_9_ | 383.1342 | 1108 | 8.04 x 10^-6^ |
| Negative | 281.0870 | 10.83 | C_10_H_17_O_9_ | 281.0873 | 19.8 | 8.19 x 10^-14c^ |
| Negative | 983.4483 | 11.03 | C_48_H_71_O_21_ | 983.4488 | 13095 | 6.65 x 10^-9^ |

^a^ Fold change indicates the concentration increase in roots colonised with AMF when compared to the concentrations observed in control plants.

^b^ Significance determined using t-tests (^c^) or Mann-Whitney U tests (unmarked) after Bonferroni corrections.

^d^ Putative identity hexadecenoyl-glycero-phosphoethanolamine.

^e^ Putative identity conjugated blumenol metabolite.

### Note 1. Identification of metabolites associated with R. irregularis colonisation

Metabolites 2 and 5 in Table S2 were identified as glycoside conjugates of blumenol C, with the same molecular ion and fragmentation patterns but with different retention times. Fragmentation gave rise to an ion corresponding to the aglycone C_13_H_23_O (*m/z* 211.1698) indicating loss of a hexose moiety *(m/z* 162, C_6_H_10_O_5_) from the parent ion. Further fragmentation of the blumenol C ring resulted in the following ions which were ± 5ppm of the calculated *m/z* of 193.1591 (C_11_H_21_O) and 175.1487 (C_11_H_19_), and which have been previously reported for this structure (Schliemann *et al.*, 2006; Schliemann *et al.*, 2008). In addition, this study observed an additional fragment which was ± 5ppm of the calculated *m/z* of 135.1175 (C_10_H_15_) (see Fig. S2 a,d). Further fragmentation of metabolite 5 resulted in ions consistent with loss of the propyl side chain. Therefore metabolites 2 and 5 were identified as steroisomers of Blumenol C glycoside with different structures or configurations of the hexose moiety (Fig. 3). Without further chemical analysis using nuclear magnetic resonance spectroscopy (NMR) the exact nature of the hexose conjugates could not be determined. Metabolite 7 was identified as blumenol C conjugated with malonylglycoside (Table S2, Fig. 3). This structure was determined by the observation of a loss of the malonyglycoside moiety (*m/z* 248, C_9_H_12_O_8_) from the parent ion, and fragmentation consistent with the blumenol C aglycone structure (Table S2).

Metabolite 6 was identified as blumenol C conjugated to a malonylglycosyl and uronide moiety, a unique metabolite hitherto unreported (Table S2, Fig. 3). Metabolite 6 was detected as the same metabolite, with the same retention time, in both positive and negative ESI modes. Fragmentation of the molecular ion in positive mode resulted in a fragment of m/z 459.2230 which was ± 5ppm of the calculated *m/z* 459.2230 and corresponded to an elemental composition C_22_H_35_O_10_. The 459 ion corresponded to the loss of C_6_H_8_O_6_ from the parent ion and indicated the loss of a conjugate of the elemental composition C_6_H_10_O_7,_ an uronic acid assuming loss of a water molecule during conjugation. This group was most likely to be glucuronic acid as most blumenol structures identified to date are conjugated to either glucose or glucuronic acid or malonic acid groups (Strack and Fester, 2006). Further fragmentation gave rise to ions corresponding to the unconjugated blumenol C ring (*m/z*: 211.1698, elemental composition: C_13_H_23_O_2_), which indicated that metabolite 6 was also conjugated to a malonylglycoside group (C_9_H_12_O_8_). The order of conjugate loss demonstrated that the glucuronic acid was probably conjugated to the glycosylmalonyl group. The fragmentation pattern observed in negative ESI, along with their elemental composition, agreed with the structure identified from the positive ESI data. A key fragment of m/z 371.2072 corresponded to loss of malonyl glucuronide from blumenol C glycoside, further supporting conjugation of the glucuronide moiety to the malonyl group (Table S2). However, further analytical work would be required to confirm the position of the glucuronic acid on the glycoside structure.

Metabolite 3 was identified as blumenol C glycosylglucuronide (Maier *et al.*, 1995) and was detected in negative ESI mode (Table S2, Fig. 2). Fragmentation of the molecular ion resulted in a fragment ± 5ppm of the calculated *m/z* 175.0243 (C_6_H_7_O_6_) which represented a conjugate with the elemental composition C_6_H_10_O_7_ (assumed to be glucuronic acid) supposing loss of a water molecule during conjugation. Further fragmentation of the molecular ion gave rise to ions corresponding to the unconjugated blumenol C ring (*m/*z: 209.1542, elemental composition: C_13_H_21_O_2_), which indicated that metabolite 3 was also conjugated with a glycoside group (C_6_H_11_O_5_) assuming the loss of a hydroxyl group during conjugation. The order of conjugate loss suggested that the glucuronic acid was conjugated to the glycosyl group.

A second group of conjugated blumenols were identified that contained an aglycone composition of C_13_H_23_O_3_ (13-hydroxyblumenol C), and these were metabolites 1 and 4 in Table S2. Fragmentation of metabolite 1 in positive ESI mode gave rise to an ion which was ± 5ppm of the calculated *m/z* of 227.1647 and corresponded to a C_13_H_23_O_3_ blumenol ring structure. The loss of *m/z* 162 (C_6_H_10_O_5_) from the parent ion indicated that the structure was a glycoside conjugate. Further fragmentation of the aglycone structure resulted in ions that were ± 5ppm of the calculated *m/z* of 209.1543 (C_13_H_21_O_2_) and 191.1440 (C_13_H_19_O) that were consistent with previous reports for this structure (Schliemann *et al.*, 2006; Schliemann *et al.*, 2008). Three additional fragment ions of the aglycone structure were present which were ± 5ppm of the calculated *m/z* of 173.1330 (C_13_H_17_) 163.1484 (C_12_H_19_) and 149.0940 (C_10_H_13_O). The fragment of *m/z* 163.1484 (C_12_H_19_) corresponded to the loss of CH_2_OH from the aglycone structure and indicated that the aglycone was 13-hydroxyblumenol C rather than a blumenol B type structure (see Fig. S2b,c,e). Metabolite 1 was also observed in negative ESI, where the calculated empirical formula was also consistent with the identified structure of 13-hydroxyblumenol C glycoside (Fig. 2). Metabolite 4 was identified as 13-hydroxyblumenol C malonylglycoside. This was determined by the loss of *m/z* 248 (C_9_H_12_O_8_) from the parent ion leaving the aglycone 13-hydroxyblumenol C (*m/z*: 277.1647, elemental composition: C_13_H_23_O_2_) (Table S2, Fig. 2).

The remaining identified metabolite was not an apocarotenoid, and metabolite 8 was identified as the lysophospholipid hexadecenoyl-glycerophosphocholine. Fragmentation of the parent ion of metabolite 8 in positive ESI mode gave rise to an ion which was ± 5ppm of the calculated *m/z* of 476.3141 and corresponded to the loss of a water molecule. Further fragmentation of the metabolite resulted in ions that were ± 5ppm of the calculated *m/z* 311.2856 (C_19_H_35_O_3_) and 184.0739 (C_5_H_15_NO_4_P) that corresponded to the chain and head of the hexadecenoyl-glycerophosphocholine structure respectively.

### PAs associated with Rhizophagus irregularis colonisation

The concentrations of four root PAs significantly increased as a result of *Rhizophagus irregularis* colonisation (Table 4). The elemental composition of the PA of *m/z* 336.1811 (C_18_H_25_NO_5_) indicated a structure corresponding to the structures of either senecivernine, integerrimine or senecionine which separate on reverse phase HPLC (Macel *et al.*, 2004; Joosten *et al.*, 2010; Kostenko *et al.*, 2012). However, the PA observed in this study, co-eluted with a senecionine standard (Carl Roth GmbH & Co, Karlsruhe, Germany) and thus it was tentatively identified as senecionine, although further analytical work would be needed to be confirm the PA structure. The PA of *m/z* 368.1716 comprised an elemental composition C_18_H_26_NO_7_, corresponding to either jacobine N-oxide, usaramine N-oxide or retrorsine N-oxide (Macel *et al.*, 2004; Joosten *et al.*, 2010; Kostenko *et al.*, 2012). UPLC-QTOFMS analysis of a retrorsine N-oxide standard (PhytoLab GmbH & Co. KG, Nürnberg, Germany) revealed that it eluted 0.4 mins later than the PA of interest. Collision-induced dissociation of *m/*z 368.1716 resulted in an ion of m/z 188.0711 corresponding to a fragment of C_11_H_10_NO_2_, and the assigned structure using predicted fragmentation software (MassFragment, Waters, Ltd) was consistent with a fragment arising from usaramine N-oxide rather than jacobine N-oxide. The PA of *m/z* 368.1815 was identified as jacoline N-oxide (C_18_H_28_NO_8_), as it is the only ragwort PA of this elemental composition (Macel *et al.*, 2004; Joosten *et al.*, 2010; Kostenko *et al.*, 2012). The PA of *m/z* 404.1476 has the characteristic isotopic pattern of a chlorinated compound (C_18_H_26_NO_7_Cl) and was identified as jaconine N-oxide (Macel *et al.*, 2004; Joosten *et al.*, 2010; Kostenko *et al.*, 2012).

.

## REFERENCES

Joosten L, Mulder PPJ, Vrieling K, van Veen JA, Klinkhamer PGL (2010) The analysis of pyrrolizidine alkaloids in *Jacobaea vulgaris*; a comparison of extraction and detection methods. Phytochem Anal 21: 197-204.

Kostenko O, van de Voorde TFJ, Mulder PPJ, van der Putten WH, Bezemer TM (2012) Legacy effects of aboveground-belowground interactions. Ecol Letts 15: 813-821.

Macel M, Vrieling K, Klinkhamer PGL (2004) Variation in pyrrolizidine alkaloid patterns of *Senecio jacobaea*. Phytochemistry 65: 865-873.

Maier W, Peipp H, Schmidt J, Wray V, Strack D (1995) Levels of a terpenoid glycoside (blumenin) and cell wall-bound phenolics in some cereal mycorrhizas. Pl Physiol 109: 465-470.

Schliemann W, Kolbe B, Schmidt J, Nimtz M, Wray V (2008) Accumulation of apocarotenoids in mycorrhizal roots of leek (*Allium porrum*). Phytochemistry 69: 1680-1688.

Schliemann W, Schmidt J, Nimtz M, Wray V, Fester T, Strack D (2006) Accumulation of apocarotenoids in mycorrhizal roots of *Ornithogalum umbellatum*. Phytochemistry 67: 1196-1205.

Strack D, Fester T. (2006) Isoprenoid metabolism and plastid reorganization in arbuscular mycorrhizal roots. New Phytol 172: 22-34.


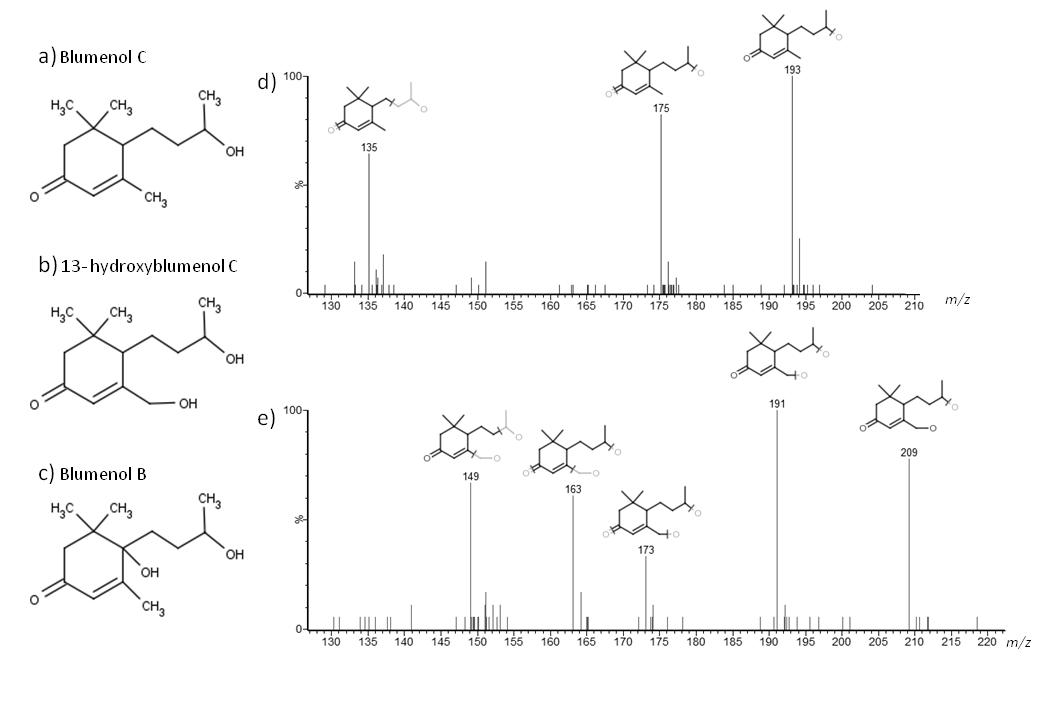


FIG S2 STRUCTURES OF A) BLUMENOL C AND B) 13-HYDROXYBLUMENOL C AND C) BLUMENOL B. THE CHARACTERISTIC FRAGMENT PATTERN ASSOCIATED WITH THE STRUCTURES OF D) BLUMENOL C AND E) 13-HYDROXYBLUMENOL C.

Spectra were recorded using UPLC-QTOFMS CID fragmentation in +ESI mode.

TABLE S3 DETAILS OF THE FRAGMENTS (OBTAINED WITH UPLC-QTOFMS CID ANALYSES) USED TO IDENTIFY THE METABOLITES THAT SIGNIFICANTLY INCREASED IN RAGWORT ROOTS COLONISED BY *RHIZOPHAGUS IRREGULARIS*

| Metabolite structure (see Fig 2.) | Observed ion (*m/z*) | UPLC- TOFMS retention time | Putative formula | Theoretical mass of ion | Δ PPM* | *m/z* of additional ions and fragments | Formula of ion | Putative identity |
| --- | --- | --- | --- | --- | --- | --- | --- | --- |
| 1 | 389.2177 | 6.37 | C_19_H_33_O_8_ | 389.2175 | 0.5 | 411.1995 | C_19_H_32_O_8_Na | 13-hydroxyblumenol C glycoside [M+H]^+^ |
|  |  |  |  |  |  | 227.1647 | C_13_H_23_O_3_ |  |
|  |  |  |  |  |  | 209.1543 | C_13_H_21_O_2_ |  |
|  |  |  |  |  |  | 191.1440 | C_13_H_19_O |  |
|  |  |  |  |  |  | 173.1330 | C_13_H_17_ |  |
|  |  |  |  |  |  | 163.1484 | C_12_H_19_ |  |
|  |  |  |  |  |  | 149.0940 | C_10_H_13_O |  |
| 1 | 387.2018 | 6.37 | C_19_H_33_O_8_ | 387.2018 | -0.3 | 433.2072 | C_20_H_34_O_10_ | 13-hydroxyblumenol C glycoside [M-H]^-^ |
| 2 | 373.2228 | 7.23 | C_19_H_33_O_7_ | 373.2226 | 0.5 | 211.1698 | C_13_H_23_O_2_ | Blumenol C glycoside [M+H]^+^ |
|  |  |  |  |  |  | 193.1591 | C_13_H_21_O |  |
|  |  |  |  |  |  | 175.1487 | C_13_H_19_ |  |
|  |  |  |  |  |  | 135.1175 | C_10_H_15_ |  |
| 3 | 547.2389 | 7.30 | C_25_H_39_O_13_ | 547.2391 | -0.4 | 429.2125 | C_21_H_33_O_9_ | Blumenol C glycosyl-glucuronide [M-H]^-^ |
|  |  |  |  |  |  | 209.1542 | C_13_H_21_O_2_ |  |
|  |  |  |  |  |  | 175.0243 | C_6_H_7_O_6_ |  |
| 4 | 475.2182 | 7.55 | C_22_H_35_O_11_ | 475.2179 | 0.6 | 227.1647 | C_13_H_23_O_3_ | 13-hydroxyblumenol C malonylglycoside [M+H]^+^ |
|  |  |  |  |  |  | 209.1543 | C_13_H_21_O_2_ |  |
|  |  |  |  |  |  | 191.1440 | C_13_H_19_O |  |
|  |  |  |  |  |  | 173.1330 | C_13_H_17_ |  |
|  |  |  |  |  |  | 163.1484 | C_12_H_19_ |  |
|  |  |  |  |  |  | 149.0940 | C_10_H_13_O |  |

TABLE S3 CONTINUED...

| Metabolite number | Observed ion (*m/z*) | UPLC- TOFMS r.t. | Putative formula | Theoretical mass of ion | Δ PPM | *m/z* of additional ions and fragments | Formula of ion | Putative identity |
| --- | --- | --- | --- | --- | --- | --- | --- | --- |
| 5 | 373.2222 | 8.20 | C_19_H_33_O_7_ | 373.2226 | -1.1 | 395.2046 | C_19_H_32_O_7_Na | Blumenol C glycoside [M+H]^+^ |
|  |  |  |  |  |  | 211.1698 | C_13_H_23_O_2_ |  |
|  |  |  |  |  |  | 193.1591 | C_13_H_21_O |  |
|  |  |  |  |  |  | 175.1487 | C_13_H_19_ |  |
|  |  |  |  |  |  | 135.1175 | C_10_H_15_ |  |
|  |  |  |  |  |  | 109.1012 | C_8_H_13_ |  |
|  |  |  |  |  |  | 95.0821 | C_7_H_11_ |  |
| 6 | 635.2551 | 8.24 | C_28_H_43_O_16_ | 635.2551 | 0.0 | 657.2363 | C_28_H_42_O_16_Na | Blumenol C malonylglycosyl-glucuronide [M+H]^+^ |
|  |  |  |  |  |  | 595.2023 | C_28_H_35_O_14_ |  |
|  |  |  |  |  |  | 459.2230 | C_22_H_35_O_10_ |  |
|  |  |  |  |  |  | 423.2014 | C_22_H_31_O_8_ |  |
|  |  |  |  |  |  | 211.1698 | C_13_H_23_O_2_ |  |
|  |  |  |  |  |  | 193.1592 | C_13_H_21_O |  |
|  |  |  |  |  |  | 175.1489 | C_13_H_19_ |  |
|  |  |  |  |  |  | 135.1174 | C_10_H_15_ |  |
|  |  |  |  |  |  | 109.1021 | C_8_H_13_ |  |
| 6 | 633.2396 | 8.24 | C_28_H_41_O_16_ | 633.2395 | 0.2 | 655.2216 | C_28_H_40_O_16_Na | Blumenol C malonylglycosyl- glucuronide [M-H]^-^ |
|  |  |  |  |  |  | 589.2495 | C_27_H_41_O_14_ |  |
|  |  |  |  |  |  | 529.2285 | C_25_H_37_O_12_ |  |
|  |  |  |  |  |  | 371.2072 | C_19_H_31_O_7_ |  |
|  |  |  |  |  |  | 161.0447 | C_6_H_9_O_5_ |  |

TABLE S3 CONTINUED...

| Metabolite number | Observed ion (*m/z*) | UPLC- TOFMS r.t. | Putative formula | Theoretical mass of ion | Δ PPM | *m/z* of additional ions and fragments | Formula of ion | Putative identity |
| --- | --- | --- | --- | --- | --- | --- | --- | --- |
| 7 | 459.2232 | 9.38 | C_22_H_35_O_10_ | 459.2230 | 0.5 | 481.2050 | C_22_H_34_O_10_Na | Blumenol C malonylglycoside [M+H]^+^ |
|  |  |  |  |  |  | 437.2149 | C_21_H_34_O_8_Na |  |
|  |  |  |  |  |  | 211.1698 | C_13_H_23_O_2_ |  |
|  |  |  |  |  |  | 193.1592 | C_13_H_21_O |  |
|  |  |  |  |  |  | 175.1487 | C_13_H_19_ |  |
|  |  |  |  |  |  | 135.1174 | C_10_H_15_ |  |
|  |  |  |  |  |  | 109.1020 | C_8_H_13_ |  |
| 8 | 494.3247 | 14.20 | C_24_H_49_NO_7_P | 494.3247 | 0.0 | 476.3141 | C_24_H_47_NO_6_P | Hexadecenoyl-glycero-phosphocholine [M+H]^+^ |
|  |  |  |  |  |  | 311.2856 | C_19_H_35_O_3_ |  |
|  |  |  |  |  |  | 184.0739 | C_5_H_15_NO_4_P |  |

Δ PPM* difference between observed and calculated m/z of ion.

TABLE S4 AVERAGE RELATIVE CONCENTRATIONS (±S.E.) OF THE PYRROLIZIDINE ALKALOID (PA) SIGNALS MEASURED IN THE POSITIVE ESI UPLC-TOFMS PROFILES OF CONTROL (N=15) AND *RHIZOPHAGUS IRREGULARIS* COLONISED (N=16) RAGWORT LEAVES

| Theoretical mass of ion | Putative formula | UPLC-TOFMS r.t. | | New leaves | | | | | |  | | Old leaves | | | | | |  |
| --- | --- | --- | --- | --- | --- | --- | --- | --- | --- | --- | --- | --- | --- | --- | --- | --- | --- | --- |
|  |  |  |  | Control | | With AM fungi | | P-value^a^ | |  | | Control | | With AM fungi | | P-value^a^ | |  |
| 334.1654 | C_18_H_23_NO_5_ | | 6.00 | | 110.8 (±11.9) | | 103.0 (±12.1) | | 0.654 | |  | | 39.1 (±7.1) | | 47.3 (±10.7) | | 0.953 | |
| 336.1811 | C_18_H_25_NO_5_ | | 6.86 | | 213.1 (±34.5) | | 168.4 (±25.9) | | 0.299 | |  | | 54.8 (±11.8) | | 84.2 (±17.8) | | 0.188 | |
| 350.1604 | C_18_H_23_NO_6_ | | 4.10 | | 20.0 (±6.3) | | 16.5 (±5.3) | | 0.711 | |  | | 29.3 (±12.0) | | 64.6 (±18.3) | | 0.078 | |
|  | and isomers of | | 6.34 | | 741.2 (±75.5) | | 762.9 (±83.4) | | 0.953 | |  | | 135.4 (±33.3) | | 198.6 (±51.7) | | 0.572 | |
|  |  | | 9.05 | | 71.4 (±18.2) | | 40.1 (±12.3) | | 0.401 | |  | | 11.3 (±5.5) | | 7.4 (±2.1) | | 0.953 | |
| 352.1760 | C_18_H_25_NO_6_ | | 4.90 | | 114.4 (±54.7) | | 140.0 (±61.7) | | 0.626 | |  | | 769.0 (±351.0) | | 1758.0 (±475.3) | | 0.024 | |
|  | and isomers of | | 5.42 | | 20.3 (±5.5) | | 15.3 (±3.1) | | 0.545 | |  | | 4.4 (±1.9) | | 1.3 (±0.5) | | 0.041 | |
|  |  | | 6.26 | | 132.1 (±14.3) | | 129.8 (±11.2) | | 0.830 | |  | | 40.7 (±8.1) | | 46.8 (±9.7) | | 0.892 | |
|  |  | | 7.01 | | 854.3 (±170.4) | | 680.7 (±117.8) | | 0.682 | |  | | 168.1 (±48.7) | | 238.5 (±60.3) | | 0.247 | |
| 366.1553 | C_18_H_23_NO_7_ | | 4.28 | | 467.8 (±59.0) | | 469.9 (±77.1) | | 0.740 | |  | | 30.9 (±5.2) | | 29.8 (±5.5) | | 0.800 | |
|  | and isomers of | | 4.97 | | 119.7 (±33.1) | | 138.2 (±34.4) | | 0.711 | |  | | 26.4 (±10.9) | | 20.9 (±7.9) | | 0.922 | |
|  |  | | 6.79 | | 76.8 (±19.5) | | 144.6 (±33.8) | | 0.086 | |  | | 11.8 (±4.8) | | 23.2 (±6.5) | | 0.049 | |
| 368.1709 | C_18_H_25_NO_7_ | | 5.25 | | 326.4 (±93.8) | | 763.8 (±212.0) | | 0.101 | |  | | 72.9 (±20.0) | | 171.6 (±33.7) | | 0.027 | |
|  | and isomers of | | 5.65 | | 181.9 (±50.3) | | 158.4 (±30.3) | | 0.922 | |  | | 155.5 (±80.8) | | 86.7 (±24.6) | | 0.830 | |
|  |  | | 6.40 | | 33.4 (±5.3) | | 28.6 (±3.4) | | 0.740 | |  | | 34.3 (±3.6) | | 33.0 (±5.5) | | 0.379 | |
| 386.1815 | C_18_H_27_NO_8_ | | 3.69 | | 24.6 (±8.6) | | 59.6 (±18.0) | | 0.037 | |  | | 23.1 (±6.2) | | 66.2 (±12.9) | | 0.019 | |
| 404.1476 | C_18_H_26_NO_7_Cl | | 5.24 | | 11.4 (±3.8) | | 27.6 (±3.8) | | 0.011 | |  | | 17.8 (±4.9) | | 47.7 (±9.5) | | 0.027 | |

^a^ No P-values remain significant after Benjamini and Hochberg adjustment.

Relative concentrations were quantified as the ratio of the analyte signal: internal standard signal per 0.3 mg plant mass.
